# Impact of Perovskite Composition on Film Formation Quality and Photophysical Properties for Flexible Perovskite Solar Cells

**DOI:** 10.3390/molecules25030732

**Published:** 2020-02-07

**Authors:** Guangdong Li, Xiaoping Zou, Jin Cheng, Dan Chen, Yujun Yao, Chuangchuang Chang, Xing Yu, Zixiao Zhou, Junqi Wang, Baoyu Liu

**Affiliations:** 1 Beijing Advanced Innovation Center for Materials Genome Engineering, Research Center for Sensor Technology, Beijing Key Laboratory for Sensor, MOE Key Laboratory for Modern Measurement and Control Technology, School of Applied Sciences, Beijing Information Science and Technology University, Jianxiangqiao Campus, Beijing 100101, China; lgd1511455720@163.com (G.L.); chengjin@bistu.edu.cn (J.C.); yyj10zy@gmail.com (Y.Y.); changcc037@gmail.com (C.C.); nimingyx1@163.com (X.Y.); zzxfpp111@163.com (Z.Z.); 13126706081@163.com (J.W.); liubaoyu0214@163.com (B.L.); 2 State Key Laboratory on Integrated Optoelectronics, Institute of Semiconductors, Chinese Academy of Sciences, Beijing 100083, China; chendan1988@semi.ac.cn; 3 Center of Materials Science and Optoelectronics Engineering, University of Chinese Academy of Sciences, Beijing 100049, China

**Keywords:** perovskite composition, film quality, photophysical properties, flexible perovskite solar cells

## Abstract

In recent years, flexible perovskite solar cells have drawn tremendous attention in the field of wearable devices, and optimization of perovskite composition plays an important role in improving film quality and photophysical properties. At present, some researchers have only studied A-site organic cations mixing or X-site halide anions mixing in the ABX_3_ structure of perovskite, but there are few reports on co-mixing of A-site and X-site ions in flexible perovskite solar cells. In this paper, we mainly try to study the effects of different concentrations of mixed formamidine methylamine halide (FA_x_MA_1-x_Br_x_Cl_y_I_1-x-y_) precursor solutions on the quality and photophysical properties of perovskite films under low temperature process. We conclude that the film quality and photophysical properties reached the best results when the optimized precursor solution concentration was 60:6:6. The investigation on composition optimization in this experiment laid the foundation for the improvement of the performance of flexible perovskite solar cells. We also use the results of this experiment to prepare flexible perovskite solar cells based on carbon electrodes, which are expected to be applied in other flexible optoelectronic or electro-optical devices.

## 1. Introduction

Flexible perovskite solar cells are expected to be integrated into wearable mobile devices to form self-powered devices due to their light weight, flexibility, and low cost, which has aroused increasing interest in the field of organic–inorganic hybrid perovskite solar cells [1,2,3,4,5]. Recently, the power conversion efficiency (PCE) of flexible perovskite solar cells has rocketed from the initial 6% to 18.4% [6,7].

There are many factors that affect the quality and photophysical properties of perovskite films. For example, the different composition of the perovskite functional layer will change the quality and photophysical properties of the perovskite film. The concentration of the precursor solution has a greater effect on the morphology of the perovskite layer, which affects the efficiency of the device [8,9,10]. In 2017, Wang et al. prepared flexible perovskite solar cells by mixing CH_3_NH_3_^+^ (MA^+^) and HN=CHNH_3_^+^ (FA^+^), extending the absorption edge of perovskite to longer wavelength, enhancing photocurrent of the flexible perovskite solar cells, and significantly improving the performance of the device [11]. In 2018, Du et al. prepared flexible perovskite solar cell with a PCE of 13.27% by mixing I^-^ and Cl^-^ in perovskite, and adding suitable H_2_O additive to the perovskite precursor solution, improving film quality [12].

However, when FA^+^ and MA^+^ are mixed in the perovskite, grains become much smaller, leading to relatively high grain boundary density, which may cause charge recombination [11]. Therefore, in our study, PC_61_BM was used to passivate the grain boundaries of perovskite film, increase the grain size, and improve device performance [11,13].

FA^+^ has the advantages of changing the lattice parameters of perovskite and reducing the perovskite band gap, and the thermal stability of solar cells can also be improved when FA^+^ and MA^+^ are mixed [11,14]; Cl^-^ and Br^−^ improve the crystallinity and coverage of the films [15]. In recent years, for flexible perovskite solar cells, there have been a few reports on the mixing of organic cations or halide anions in perovskite layers [11,12], but there have been few reports on the co-mixing of organic cations and halide anions. Therefore, based on the above advantages, different from previous studies, this experiment was oriented to flexible perovskite solar cells. We prepared mixed HC(NH_2_)_2_)_x_(CH_3_NH_3_)_1-x_PbBr_x_Cl_y_I_3-x-y_/FA_x_MA_1-x_PbBr_x_Cl_y_I_3-x-y_ perovskite by using FA^+^ and MA^+^ to achieve the A-site mixing in the typical ABX_3_ structure of perovskite and by using I^−^, Cl^−^, and Br^−^ to achieve X-site mixing. By changing the concentration of the mixed formamidine methylamine halide (FA_x_MA_1-x_Br_x_Cl_y_I_1-x-y_) precursor solution, we prepared perovskite solar cells under low temperature process and characterized the perovskite film and device performance. We have also obtained an optimal ratio of precursor solution, which also has laid the foundation for the flexible perovskite solar cell preparation and optimization process; we then used the results of this experimental study to prepare flexible perovskite solar cells based on carbon film counter electrodes.

In this experiment, we prepared perovskite layers by adjusting the concentrations of mixed formamidine methylamine halide (FA_x_MA_1-x_Br_x_Cl_y_I_1-x-y_) precursor solution, and then prepared the perovskite solar cell. Through the characterization of the perovskite film and device performance, we found that when the concentration was 60:6:6, the film quality, photophysical properties, and device performance reached the best result.

## 2. Results and Discussion

**Figure 1:**
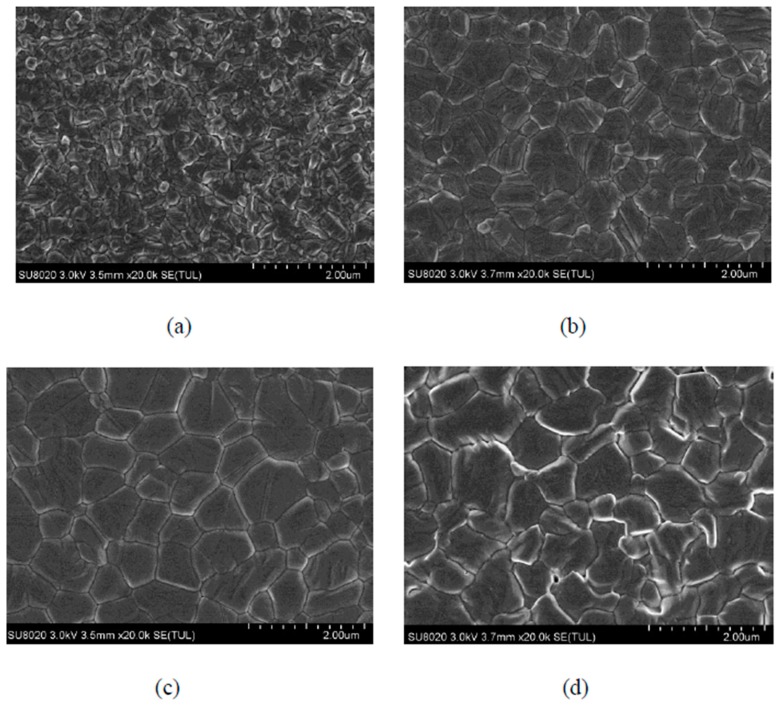
SEM images of perovskite layer prepared with formamidine methylamine halide precursors solutions with concentrations of 40:4:4 (**a**), 50:5:5 (**b**), 60:6:6 (**c**), and 70:7:7 (**d**), respectively.

In this paper, we mainly investigated the effects of different concentrations of mixed formamidine methylamine halide (FA_x_MA_1-x_Br_x_Cl_y_I_1-x-y_) precursor solution on perovskite film quality and photophysical properties under a low temperature process. SEM images of perovskite layers prepared with different concentrations of FA_x_MA_1-x_Br_x_Cl_y_I_1-x-y_ precursor solutions are shown in Figure 1. Figure 1 are SEM images of perovskite layers prepared at concentrations of precursor solutions of 40:4:4, 50:5:5, 60:6:6, and 70:7:7, respectively, at a magnification of 20,000 times.

It can be seen from Figure 1a that when the concentration of FA_x_MA_1-x_Br_x_Cl_y_I_1-x-y_ precursor solution was 40:4:4, the size of the perovskite grain was small, and the shape was irregular. As seen in Figure 1b, when the concentration of the solution was 50:5:5, the grain size of perovskite increased, and the grain boundary was clearer, but there were traces of scale growth on the surface of the grains. When the concentration of the solution was increased to 60:6:6, compared with Figure 1a,b as shown in Figure 1c, the diameter of the perovskite grains reached the maximum value, and the largest grain diameter even exceeded 1 μm. The film surface was flat, the shape was regular15, the grain boundaries were clear, and there were no obvious defects; thus, the film quality was better. As seen in Figure 1d, when the concentration of the solution continued to increase to 70:7:7, obvious pinholes appeared on the surface of the film, the grain boundary became blurred, and the film surface became rough, which may have been caused by the abnormal growth of perovskite [16]. Based on the above analysis, we drew the conclusion that the quality of perovskite thin film first got better and then worse with the increase of the concentration of mixed FA_x_MA_1-x_Br_x_Cl_y_I_1-x-y_ precursor solution. The film quality was best when the precursor solution concentration was 60:6:6.

**Figure 2:**
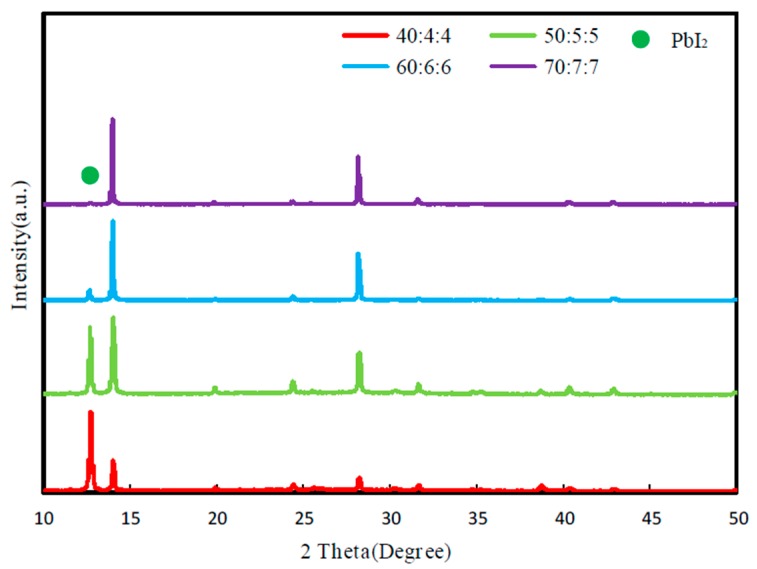
Normalized XRD patterns of perovskite layers prepared with mixed formamidine methylamine halide precursor solutions at different concentrations.

Figure 2 shows the normalized XRD patterns of perovskite layer prepared with FA_x_MA_1-x_Br_x_Cl_y_I_1-x-y_ precursor solution of different concentrations. The peak at 2θ at 12.5° was the characteristic peak of PbI_2_. The diffraction peaks at 14.0° and 28.1° corresponded to the (111) and (222) planes of the perovskite, and the diffraction peak at 31.6° was the (123) plane of the perovskite crystals [17]. It was found that when the concentration of the precursor solution was 40:4:4, the ratio of the PbI_2_ emission peak (12.5°) to the perovskite emission peak (14.0°) was greater than 2, which indicated the ratio of the two precursor solutions mixed to form perovskite was very low. As the solution concentration increased, the emission peak of PbI_2_ gradually decreased, which means that more PbI_2_ was involved in the chemical reaction to form perovskite [9,11]. When the solution concentration was 60:6:6, there was a slight PbI_2_ residue in the crystal, which would be beneficial to passivate the grain boundary of perovskite film and improve carrier life [18,19]. The ratio of the intensity of the 28.1° diffraction peak to the 31.6° diffraction peak at this concentration was higher than that at a concentration of 70:7:7, indicating that the preferential orientation axis of the perovskite crystals prepared at a concentration of 60:6:6 was along the (111) axis, and the film had a more pure crystal phase [17], which is consistent with Figure 1c.

**Figure 3:**
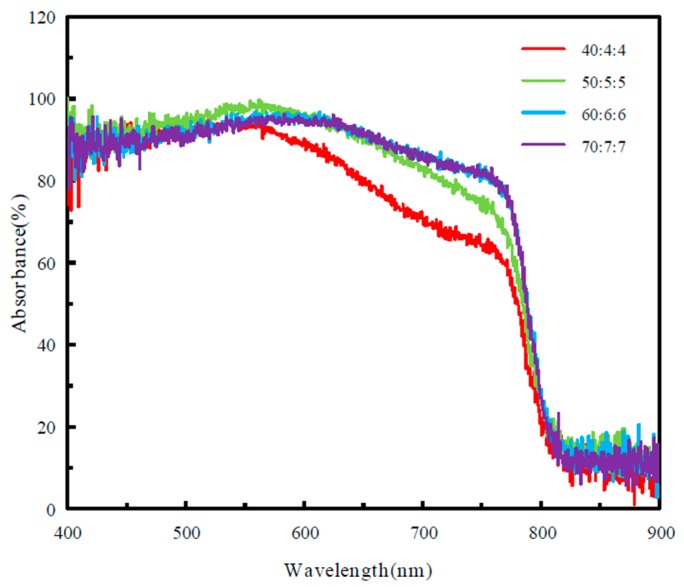
UV–vis absorption spectra of perovskite layers prepared with different concentrations of formamidine methylamine halide precursor solutions.

The UV–vis absorption spectrum of the perovskite layer prepared with FA_x_MA_1-x_Br_x_Cl_y_I_1-x-y_ precursor solution of different concentrations is shown in Figure 3, and the curve accorded with the light absorption characteristics of conventional perovskite films. When the concentration of FA_x_MA_1-x_Br_x_Cl_y_I_1-x-y_ precursor solution was 40:4:4, the UV–vis absorption spectrum of the prepared perovskite layer is shown in the red line in the figure. The absorbance of the prepared perovskite film at this concentration was lower than that at the other three concentrations, which was ascribed to the poor morphology of the film. With the increasing of concentration, the light absorption intensity increased gradually. When the precursor solution concentration was 60:6:6 and 70:7:7, the prepared perovskite films had high absorbance due to the good crystallinity of the perovskite films [20]. The perovskite film with the concentration of 40:4:4 exhibited the rough absorption edge at 801 nm; the perovskite film with the concentration of 50:5:5 exhibited the rough absorption edge at 805 nm; the perovskite film with the concentration of 70:7:7 exhibited the rough absorption edge at 808 nm; the absorption wavelength of the perovskite film prepared at the concentration of 60:6:6 was about 810 nm, which indicated that the band gap of the perovskite prepared at this concentration was narrower, which was more conducive to the generation of electron-holes after absorbing photons, improving the short-circuit current density, and thus improving the PCE of the solar cell [9].

**Figure 4:**
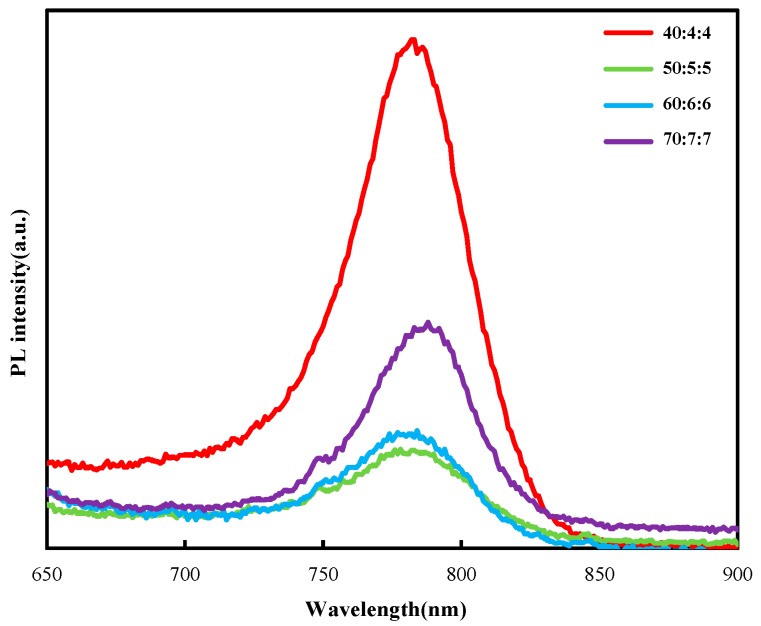
Photoluminescence spectroscopy of perovskite layers prepared with different concentrations of formamidine methylamine halide precursors solutions.

Figure 4 shows the photoluminescence spectroscopy of perovskite layers prepared by FA_x_MA_1-x_Br_x_Cl_y_I_1-x-y_ precursor solution of different concentrations. It can be seen that when the concentrations of mixed FA_x_MA_1-x_Br_x_Cl_y_I_1-x-y_ precursor solution was 50:5:5 and 60:6:6, the photoluminescence peak of the film was relatively low. This is because the two films had better film morphology, fewer defects, and lower probability of radiation recombination [12], which is also consistent with the results of the SEM diagram.

**Figure 5:**
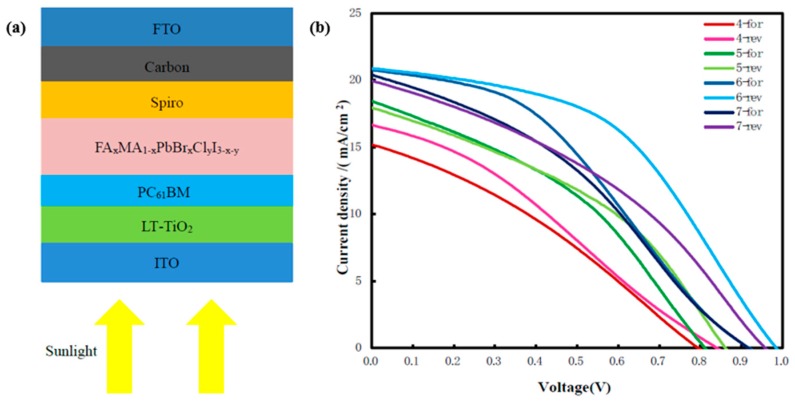
(**a**) Device structure diagram; (**b**) J–V curves of perovskite solar cells prepared with different concentrations of formamidine methylamine halide precursor solution on ITO substrate.

The device structure prepared on the indium tin oxide (ITO) conductive substrate in this experiment is shown in Figure 5a; the structures are ITO/Low-temperature (LT)-TiO_2_/PC_61_BM/FA_x_MA_1-x_PbBr_x_Cl_y_I_3-x-y_/Spiro/Carbon/Fluorine-doped tin oxide (FTO), respectively. When sunlight enters from the conductive substrate, it passes through the electron transport layer to the perovskite layer prepared by FA_x_MA_1-x_Br_x_Cl_y_I_1-x-y_ precursor solution of different concentrations, and then passes through the hole transport layer to the electrode made of carbon film. When the device is connected to the external circuit, a current is generated.

The J–V curves of perovskite solar cells prepared by FA_x_MA_1-x_Br_x_Cl_y_I_1-x-y_ precursor solution of different concentrations are shown in Figure 5b, and the performance parameters of corresponding curves in Figure 5b are shown in Table 1. In the definition diagram, 4-for represents FAI:MABr:MACl = 40 mg:4 mg:4 mg and forward scanning of the device, and 4-rev represents the reverse scanning. As the concentration of the mixed FA_x_MA_1-x_Br_x_Cl_y_I_1-x-y_ in precursor solution continued to increase, the PCE of the device increased first and then decreased. The best concentration was FAI:MABr:MACl = 60 mg:6 mg:6 mg, which was consistent with the corresponding parameters of the J–V curves, giving a V_oc_ of 0.98 V, a J_sc_ of 20.87 mA/cm^2^, an FF of 47.68%, and a PCE of 9.79%. This may have resulted from the proper mass ratio of the two precursor solutions and the complete solute reaction.

**Figure 6:**
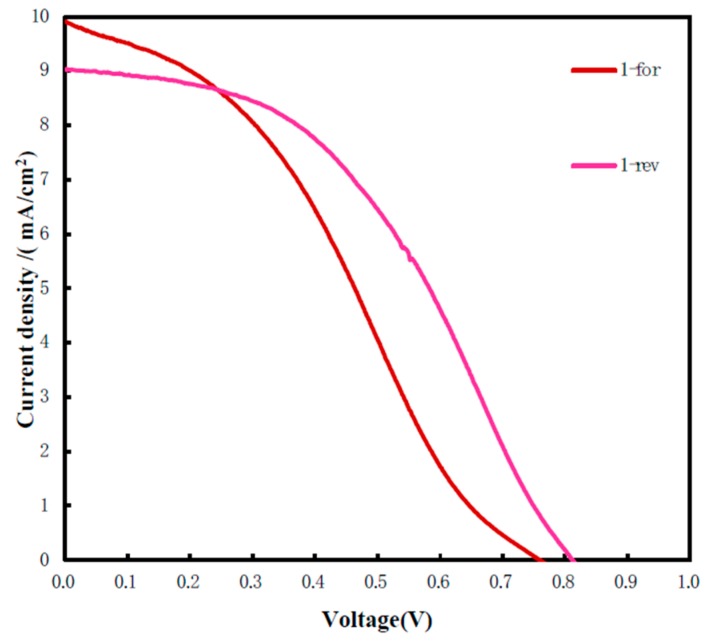
J–V curve of flexible perovskite solar cell.

**Figure 7:**
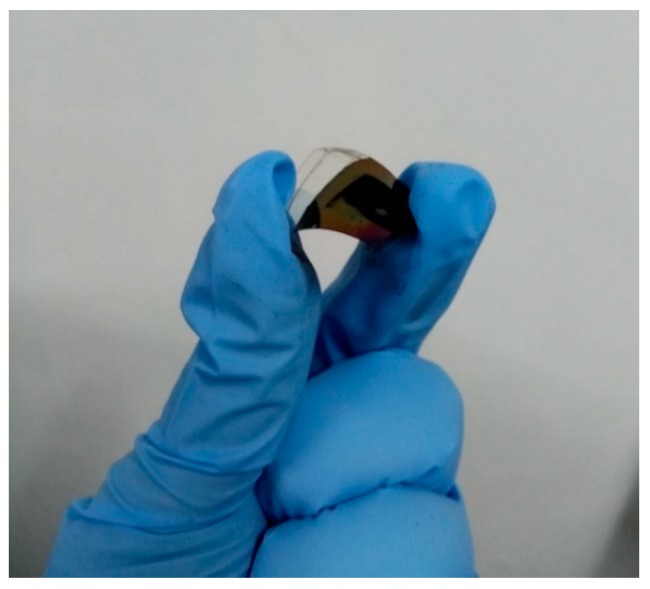
Flexible perovskite solar cell based on carbon electrode.

According to the results of the experiments discussed above, we prepared flexible perovskite solar cells on flexible ITO/polyethylene naphthalate (PEN) substrate by using the mixed FA_x_MA_1-x_Br_x_Cl_y_I_1-x-y_ precursor solution with a concentration of 60:6:6, and realized a reverse scanning PCE of 3.24%. Figure 6 shows the J–V curve of the flexible perovskite solar cell, Table 2 shows the performance parameters of the corresponding curve in Figure 6, and Figure 7 shows the photo of the flexible solar cell. In the experiment, the carbon film counter electrode was used as the solar cell photocathode and was prepared by candle flame fumigation. Due to the instability of the candle flame and the uncertainty of the fumigation time, the performance of the solar cell was greatly affected. In addition, the overall optimization of the device and the interface modification of each layer were not been completed. Later, more efforts will be made to explore better processes to improve the efficiency of flexible perovskite solar cells.

## 3. Materials and Experimental Methods

### 3.1. Materials

FTO substrate (thickness of 2.2 mm, surface resistance of 15 Ω/sq, transmittance of more than 85%) and ITO rigid substrate (thickness of 1.1 mm, surface resistance of 7 Ω/sq, about 85% light transmittance) were purchased from Shanghai Mater Win New Materials Co., Ltd. (Shanghai, China). Flexible ITO/PEN substrate (area resistance of about 15 Ω/sq) was purchased from Aoge Technology Co., Ltd. Low temperature nanocrystalline TiO_2_ spin coating solution(LT-TiO_2_), chlorobenzene (C_6_H_5_Cl) (purity ≥ 99%), dimethyl sulfoxide (DMSO) (purity ≥ 99.8%), and *N*,*N*-dimethylformamide (DMF) (purity ≥ 99.8%) were also purchased from Shanghai Mater Win New Materials Co., Ltd. (Shanghai, China). Formamidine iodine (HC(NH_2_)_2_I) (purity ≥ 99%), methylamine bromide (CH_3_NH_3_Br) (purity ≥ 99%), methylamine chloride (CH_3_NH_3_Cl) (purity ≥ 99%), lead iodide (PbI_2_) (purity ≥ 99.99%), [6,6]-phenyl C_61_ methyl butyrate (PC_61_BM) (purity ≥ 99%), and Spiro-OMeTAD were also purchased from Xi’an Polymer Light Technology Corp. (Xi’an, China).

### 3.2. Device Fabrication

The cleanliness of FTO/ITO/flexible ITO/PEN transparent conductive substrate has a very important impact on the performance of perovskite solar cells, so it must be cleaned. First, after cutting the conductive substrate and putting it into a clean petri dish, we added an appropriate amount of deionized water and detergent and placed it in an ultrasonic vibration washing machine for ultrasonic cleaning for 20 min. Next, we added an appropriate amount of absolute ethanol to the petri dish and ultrasonically cleaned it for 20 min. Then, an appropriate amount of a mixed solution of isopropanol, acetone, and deionized water was added to the petri dish, the volume ratio of which was 1:1:1, and it was ultrasonically washed for 20 min. Finally, the cleaned conductive substrate was dried at a constant temperature in a drying box for 90 min, and then placed in a UV light washer for 15 min [21].

The low temperature dense TiO_2_ layer was prepared by spin-coating low temperature nanocrystalline TiO_2_ spin coating solution on an ITO conductive substrate under a low temperature process. Only one layer was spin-coated at a spin-coating speed of 2000 rpm and a time of 30 s. First, spin-coated ITO conductive substrate was left at room temperature for 1 h, then placed on a hot plate at room temperature to heat the hot plate program to 150 °C with a heating rate of 8 °C/min, and finally at 150 °C for 20 min. The PC_61_BM electron transport layer was prepared by dissolving PC_61_BM in chlorobenzene and configuring it into PC_61_BM solution with a concentration of 20 mg/mL, and then spin-coating the solution on a low temperature dense TiO_2_ layer at a spin-coating speed of 1500 rpm and a time of 30 s.

The perovskite layer was prepared by dissolving PbI_2_ in a mixed solution of *N*,*N*-dimethylformamide (DMF) and dimethyl sulfoxide (DMSO) (the volume ratio of DMF to DMSO was 0.95:0.05) to form 600 mg/mL of precursor solution. Then the mass ratios of FAI, MABr, and MACl of 40 mg:4 mg:4 mg; 50 mg:5 mg:5 mg; 60 mg:6 mg:6 mg; and 70 mg:7 mg:7 mg, respectively, were dissolved in 1 mL of anhydrous isopropanol to prepare mixed solutions of different concentrations. Then, the mass ratios of FAI, MABr, and MACl, which were 40 mg:4 mg:4 mg; 50 mg:5 mg:5 mg; 60 mg:6 mg:6 mg; and 70 mg:7 mg:7 mg, respectively, were dissolved in 1 mL of anhydrous isopropanol to form 4 different concentrations of formamidine methylamine halide (FA_x_MA_1-x_Br_x_Cl_y_I_1-x-y_) precursor solutions. Next, the PbI_2_ precursor solution was directly spin-coated on the electron-transporting layer of PC_61_BM; the spin-coating speed was 1500 rpm, and the spin-coating time was 30 s. After the rotation was stopped, the mixed methylformamide methylamine halide (FAxMA_1-x_Br_x_ClyI_1-x__-y_) precursor solution with different concentrations was evenly drip-coated on the unheated PbI_2_ film using a pipette and then spin-coated immediately with a spin-coating speed of 1500 rpm; the spin coating time was 30 s. Finally, the spin-coated substrate was immediately placed on a glue dryer and heated at 150 °C for 20 min. In this way, perovskite film was made.

The hole-transporting layer was spin-coated on a perovskite film with Spiro-OMeTAD solution at a speed of 3000 rpm for 30 s [22]. Finally, the conductive surface of the cleaned FTO conductive glass substrate was cleaned with a candle flame to make a carbon film counter electrode. The carbon film was aligned on the top of the device, and the two sides were clamped with clips to complete the device preparation [23].

### 3.3. Characterization

A field emission scanning electron microscope (SEM) (SIGMA, Zeiss, Jena, Germany) was used to characterize the perovskite film. An XRD spectrum was obtained using an X-ray diffractometer (D8 Focus, Bruker, Dresden, Germany) from a perovskite film deposited on ITO/LT-TiO_2_/PC_61_BM. The absorption spectra of perovskite films were obtained by an ultraviolet (UV) visible absorption spectrometer (Avantes, Apeldoorn, the Netherlands), and the photoemission spectra (PL spectra) of perovskite films were measured by a fluorescence spectrometer (HORIBA Jobin Yvon, Paris, France). Finally, a solar simulator (Sol 3A, Oriel, New port, RI, USA) was used to measure the current–voltage (J–V) characteristic curve of the solar cell under standard AM 1.5 G light.

## 4. Conclusions

In summary, our work shows that the optimization of the perovskite composition can improve the film quality and photophysical properties. The SEM image shows that when the solution concentration is 60:6:6, the quality of the perovskite film reaches the best result. From the UV–vis absorption spectrum, it can be seen that when the solution concentration is 60:6:6, the absorbance of the film is better, and the absorption edge also reaches 810 nm, which is more conducive to improving the short-circuit current density. It can be seen from the PL spectrum that with the solution concentration increasing, the photoluminescence peak decreases first and then increases. When the solution concentration is 50:5:5 and 60:6:6, the photoluminescence peaks of the film are very low, which is more conducive for the carrier to be injected from the perovskite layer into the TiO_2_ layer. Finally, according to the J–V curve, it can be seen that when the solution concentration is 60:6:6, the device efficiency reaches the highest level.

The study of perovskite composition optimization in this experiment paves the way for the exploration of co-mixing processes in flexible perovskite solar cells, which is also expected to be used in other flexible photosensitive devices or light emitting diodes. The flexible perovskite solar cell based on a carbon electrode as a photocathode is also shown, and flexible perovskite solar cells based on a gold electrode as a photocathode are under investigation.

## Figures and Tables

**Table 1:** Performance parameters of perovskite solar cells prepared with different concentrations of formamidine methylamine halide precursor solution.

Samples	PCE (%) ^a^	V_oc_ (V) ^b^	J_sc_ (mA/cm^2^) ^c^	FF (%) ^d^
4-for	3.87	0.79	15.18	32.08
4-rev	4.28	0.84	16.66	30.56
5-for	5.70	0.81	18.43	38.10
5-rev	6.02	0.86	17.96	38.96
6-for	7.28	0.92	20.78	38.19
6-rev	9.79	0.98	20.87	47.68
7-for	6.64	0.91	20.39	35.57
7-rev	7.12	0.96	19.94	37.28

^a^ Power conversion efficiency; ^b^ Open-circuit voltage; ^c^ Short-circuit photocurrent density; ^d^ Fill factor.

**Table 2:** Performance parameters of flexible perovskite solar cell.

Samples	PCE (%) ^a^	V_oc_ (V) ^b^	J_sc_ (mA/cm^2^) ^c^	FF (%) ^d^
1-for	2.60	0.76	9.92	34.52
1-rev	3.24	0.81	9.04	44.23

^a^ Power conversion efficiency; ^b^ Open-circuit voltage; ^c^ Short-circuit photocurrent density; ^d^ Fill factor.

## Sample Availability

The data used to support the findings of this study are available from the corresponding author upon request.
